# Ready for Prime Time? Using Normalization Process Theory to Evaluate Implementation Success of Personal Health Records Designed for Decision Making

**DOI:** 10.3389/fdgth.2020.575951

**Published:** 2020-11-20

**Authors:** Selena Davis

**Affiliations:** Health Information Science, University Victoria, Victoria, BC, Canada

**Keywords:** personal health records, shared decision making, self-management, patient-centric services, implementation science, normalization process theory, eHealth, mixed methods

## Abstract

Personal health records designed for shared decision making (SDM) have the potential to engage patients and provide opportunities for positive health outcomes. Given the limited number of published interventions that become normal practice, this preimplementation evaluation of an integrated SDM personal health record system (e-PHR) was underpinned by Normalization Process Theory (NPT). The theory provides a framework to analyze cognitive and behavioral mechanisms known to influence implementation success. A mixed-methods investigation was utilized to explain the work required to implement e-PHR and its potential to integrate into practice. Patients, care providers, and electronic health record (EHR) and clinical leaders (*n* = 27) offered a rich explanation of the implementation work. Reliability tests of the NPT-based instrument negated the use of scores for two of the four mechanisms. Participants indicated that e-PHR made sense as explained by two qualitative themes: game-changing technology and sensibility of change. Participants appraised e-PHR as explained by two themes: reflecting on value and monitoring and adapting. The combined qualitative and quantitative results for the other two NPT mechanisms corroborated. Participants strongly agreed (score = 4.6/5) with processes requiring an investment in commitment, explained by two themes: sharing ownership of the work and enabling involvement. Weak agreement (score = 3.6/5) was observed with processes requiring an investment in effort, explained by one theme: uncovering the challenge of building collective action, and three subthemes: assessing fit, adapting to change together, and investing in the change. Finally, participants strongly agreed (score = 4.5/5) that e-PHR would positively affect engagement in self-management decision-making in two themes: care is efficient, and care is patient-centered. Overall, successful integration of e-PHR will only be attained when systemic effort is invested to enact it. Additional investigation is needed to explore the collective action gaps to inform priorities and approaches for future implementation success. This research has implications for patients, care providers, EHR vendors, and the healthcare system for improving the effectiveness and efficiency of patient-centric services. Findings confirm the usefulness of NPT for planning and understanding implementation success of PHRs.

## Introduction

Healthcare systems and clinician practices are actively seeking health information technologies (HITs) that engage patients in decision-making as part of health self-management (1). One patient-facing HIT is the personal health record (PHR). PHRs are electronic health records (EHRs) controlled, shared, and maintained by patients to support patient-centered care (2). For optimal engagement, PHRs offer patients:

- access to their health information, such as results, clinical notes, and self-management information such as standard forms, educational materials, and protocol information in a linked or embedded knowledge base;
- the ability to contribute patient-generated data to their health record, such as subjective experience data and objective data related to their condition over time;
- health management and decision support tools, such as disease-tracking tools, goal setting, decision aids, and evidence-based reminders and alerts; and
- the means to communicate with their care providers and community support groups using mechanisms such as secure messaging and video tools (3).

These PHR characteristics were also identified as components leading to improved health outcomes for patients in a systematic review (*n* = 23), which examined conditions potentially sensitive to the PHR (4). Patients' experiences with accessing their PHR are often positive and offer feelings of empowerment and engagement (5). Further, use of a PHR improves communications, partnership with care providers, and a sense of self-management (6). But PHRs have not seen widespread adoption or impact, often a result of lacking system functionalities (typically only simple messaging, viewing results, and appointment scheduling) or limiting architecture [architected as standalone or tethered to a specific provider EHR (7, 8)], as well as lack of provider acceptance (9). Designed for function and cohesive with the broader digital health ecosystem, PHRs present an opportunity for improvement in patient engagement in self-management and decision-making.

Shared decision making (SDM) between a patient and care provider is a collaborative process resulting in a treatment decision and care plan at a specific point in time that combines the best available evidence and patient values and preferences (10). The process of SDM is modeled, based on the work of several authors (11–13), to include four core elements: (a) awareness that a decision is needed, and choice exists—acknowledge; (b) receive and interpret options, including benefits and risks—consider; (c) explore preferences, values, and goals and incorporate them into the making of the decision—decide; and (d) record the decision and track outcomes—act. The fourth SDM element, act, adapts and extends the SDM model identified by Elwyn et al. (11), to make explicit the recording of the shared decision in the patient care plan with follow-up to ensure the treatment decision respects patient preferences and to track outcomes of the decision. SDM is neither about convincing the patient to follow a care provider's recommendation nor about leaving a patient to decide on her/his own (14). When patients are more informed and empowered and participate with their care providers in making treatment decisions, they have better health outcomes (15). SDM is fundamental to patient-centered care, increases patients' and providers' satisfaction, improves quality of life, and fosters a better patient–provider relationship (16), yet it has been difficult to implement into clinical practice (13). PHRs are a promising technology for overcoming barriers for integrating SDM (7, 17).

To successfully implement a PHR designed to enable SDM, a preimplementation evaluation is useful (18) since the literature provides little guidance on the complex process of integrating PHRs (19). For PHR implementation success, patients and providers must interact differently by reorienting treatment, management, and decisions around data transparency and patient access; providers must make use of patient-reported data and patient preferences in combination with medical evidence using a collaborative care team approach; and communication options must be enhanced using integrated HIT tools. The number of implemented sociotechnical interventions that become “normalized” is limited, i.e., fits in with the routine work of individuals and the context of practice and no longer requires additional effort (20). Normalization Process Theory (NPT) seeks to understand the cognitive and behavioral work people do, individually and collectively, to integrate a complex intervention in its social context (21). NPT holds the view that many interventions implemented in healthcare settings are subject to a complex interplay between features of the intervention itself, the actions of individuals involved in the process, and aspects of the physical and social environment in which the implementation activities are undertaken (21). There is a considerable and growing body of research that supports NPT as a useful theory for explaining processes of normalization of practices associated with complex health interventions (21–25). More recently, NPT has effectively been used to aid implementation planning (21). Its applicability to the different stages of system design life cycle and its valuable set of conceptual tools for the understanding of implementation as a dynamic process make it appealing.

For this research, NPT provides an analytic framework to explain the work of care providers and patients to integrate PHR technology designed to enable SDM (e-PHR) and to indicate the level of agreement of a successful future implementation. NPT provides four sets of mechanisms that characterize different kinds of “normalization work,” and each requires particular kinds of contributions from individuals and organizations that promote or inhibit successful implementation; these are (a) coherence—processes driven by contributions of meaning; (b) cognitive participation—processes driven by contributions of commitment; (c) collective action—processes driven by contributions of effort; and (d) reflexive monitoring—processes driven by contributions in appraisal (21). The research objectives were to describe (i) the work that patients and providers do, individually and collectively, to integrate e-PHR; and (ii) the potential for e-PHR to integrate into clinical practice to engage patients in self-management decision-making. This work builds on a prior user-centered design study (*n* = 22) in which the PHR functionality required to support the four core elements of the SDM process (e-PHR) was substantiated by patients and care providers (26).

## Materials and Methods

This mixed-methods descriptive study was conducted between January and April 2018 within community care and complementing community-level services in British Columbia, Canada. The three study groups were patients (young adults with type 1 diabetes, 18–24 years of age), healthcare providers (physicians, dietitians, and nurses), and organizational providers responsible for the design, development, implementation, or management of EHR systems (government HIT leaders/clinical directors). Recruitment strategies comprised posters, social media, direct e-mail of clinics from publicly available lists, and snowball recruitment. Purposeful and convenience sampling was used, and sample size within each study group was guided by the principle of saturation and determined when the research obtained and interpreted sufficient data to reasonably understand the phenomena. The study received ethical approval from University of Victoria (protocol no. BC17-058).

This mixed-methods investigation utilized a triangulation convergence study design, i.e., concurrently collected and equally weighted quantitative measurement instrument and practice-related outcomes survey data and qualitative semistructured individual interview data. Underpinned by NPT, the investigative approach offered a deeper level of understanding and explanation about the integration of e-PHR into clinical practice and gave a voice to the multiple participant study groups.

### e-PHR

**Figure 1:**
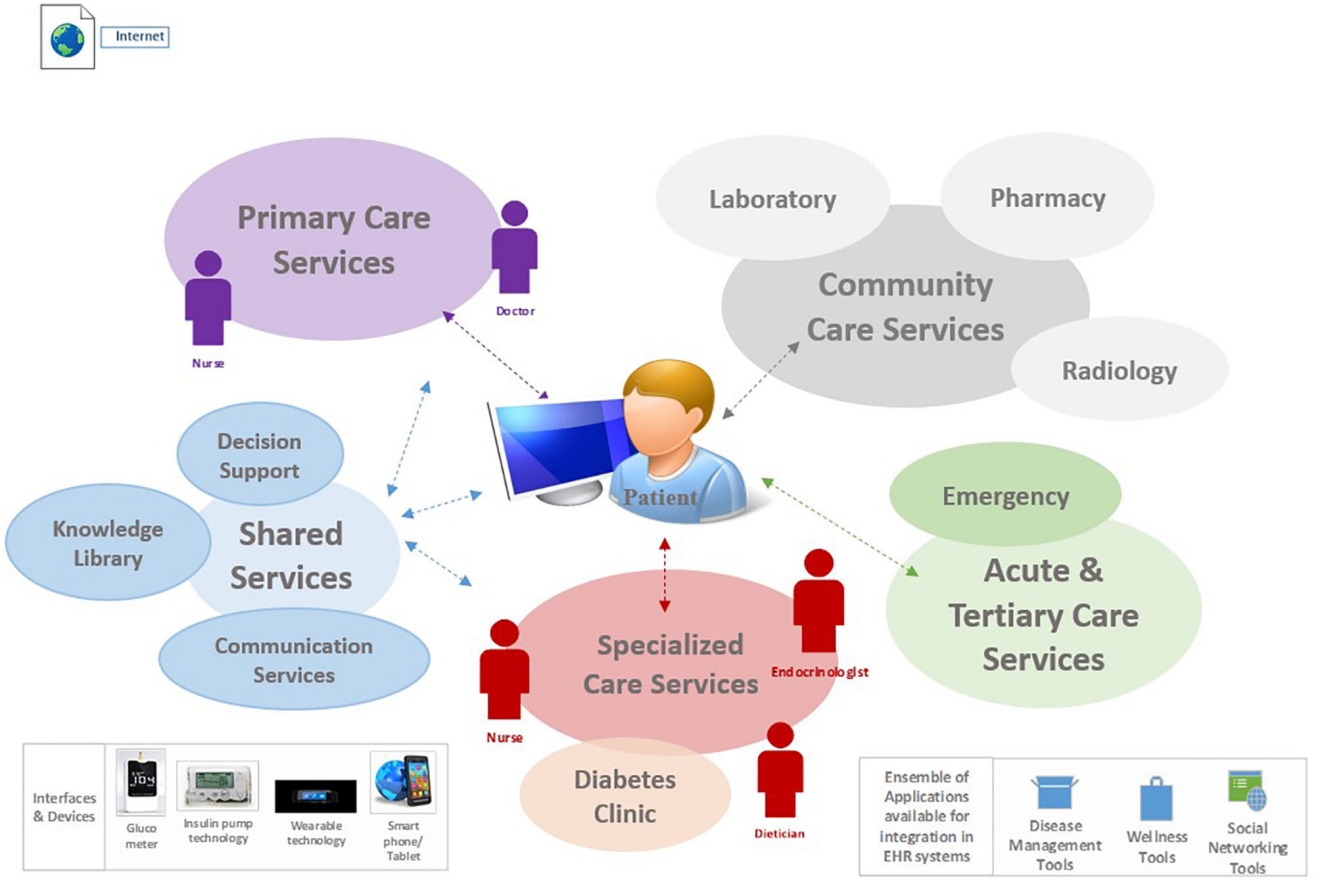
e-PHR ecosystem.

In the user-entered design study by Davis and MacKay (26), the resultant e-PHR encompassed four central PHR functionality containing 23 specific PHR functions for the enablement of SDM. According to the study (26), to enable the SDM elements *acknowledge* and *consider*, PHR functionality *receive decision-support* comprised functions such as “receive intelligent alerts,” “receive personalized decision support resources,” and “elicit preference in context of a treatment decision.” To enable the SDM element *decide*, PHR functionality *access health information* and *communicate with others* comprised functions such as “review provider clinical notes and annotated data in provider EHR” and “participate in a virtual consultation with provider.” To enable the SDM element act, PHR functionality *record health information* comprised functions such as “coauthor care plan.”

e-PHR was described by participants in the prior study (26) as one that should be architected as an interconnected PHR; i.e., it gathers and autopopulates patient data from multiple health information systems and applications. Figure 1 illustrates the ecosystem for e-PHR, contextualized from the perspective of a patient with diabetes and simplified in terms of integration with the overarching electronic healthcare information systems, including the connectivity of interfaces, devices, and applications required by patients to self-manage their health. This figure was provided to participants in this study as part of an online video prior to data collection.

### Guiding Theoretical Framework

**Figure 2:**
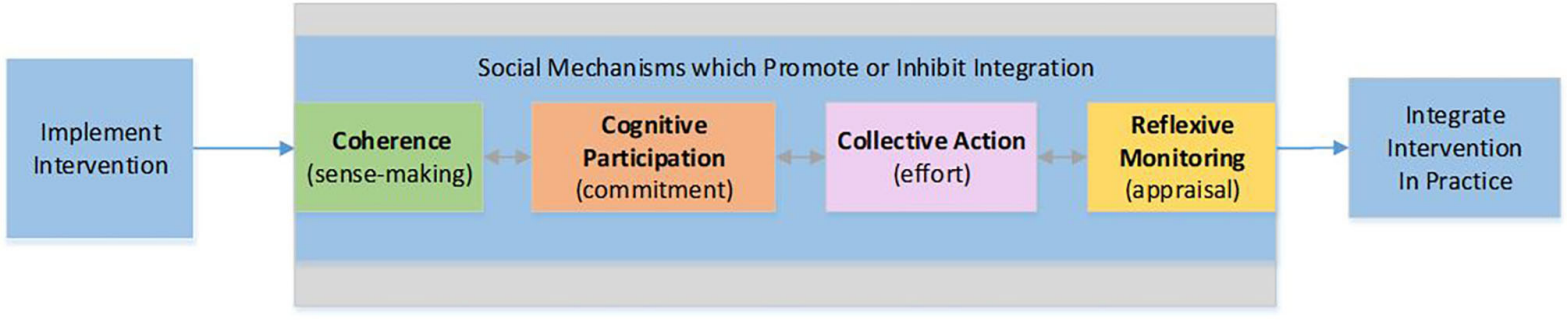
Normalization Process Theory framework.

NPT provides a framework to analyze four process mechanisms and their related constructs known to influence implementation success (Figure 2). Coherence is the sense-making work that people do individually and collectively when they are faced with the problem of operationalizing e-PHR. Cognitive participation is the relational and commitment work that individuals in teams do as they anticipate roles and tasks to accomplish new ways of doing things with e-PHR. Collective action is the operational or effort-type work needed to enact e-PHR. Reflexive monitoring is the appraisal work that people do to assess and understand the ways that e-PHR affects them and others around them.

### Data Collection

At the beginning of the participant's scheduled virtual meeting, the researcher recapped the study details, and consent was affirmed and audio recorded. Then, the participant received an e-mail with a PDF of the user-validated functional model for e-PHR (Supplementary Material 1) and a link to an online, 3-min video (https://youtu.be/mV2koq1KN58) that was created to provide participants with more details and context of e-PHR.

The quantitative data were gathered by both a measurement instrument and survey. The new and first quantitative measurement instrument of NPT, the Normalization MeAsure Development (NoMAD), was used (Supplementary Material 2) (27). The NoMAD comprises 20 NPT constructs, separated into groups representing the four NPT mechanisms (coherence, cognitive participation, collective action, and reflexive monitoring) with items rated on a 5-point Likert scale. The NoMAD was administered to describe the level of agreement of patients, care providers, and organizational leaders with statements of the four NPT mechanisms and their related constructs known to influence the integration of an intervention. For example, frequent “strongly agree” responses (Likert scores = 5) indicate the intervention “makes sense” to participants (coherence) or that specific aspects of effort (collective action) appear low, given the frequency of “strongly disagree” responses (Likert scores = 1). While NoMAD has been identified as a robust instrument for use in quantitative investigations (21), at the initiation of this study, full psychometric testing had not been completed, so basic psychometric evaluation was included as part of this study. Utilizing the same five-point Likert scale as the measurement instrument, a small fixed survey of practice-related outcomes (Supplementary Material 3) scored the level of agreement with potential outcomes such as engagement in self-management decision-making, easier to participate in SDM, and e-PHR system would normalize in clinical practice. Online self-management interventions have demonstrated increased patient engagement, an important factor in helping patients to manage their health (28). As such, this research was interested in the potential for engagement as an outcome. Both the instrument and survey were delivered online to participants via SimpleSurvey (29) and a link provided in an email.

Once the instrument and survey were submitted, the phone interview commenced for the collection of qualitative data. The semistructured interview conducted by the researcher (S.D.) followed an interview guide (Supplementary Material 4) and was structured using the four process mechanisms of NPT to describe the work of integrating e-PHR into clinical practice (Table 1).

**Table 1:** Interview questions aligned with NPT.

**Interview question**	**NPT mechanism**
How would you describe the e-PHR and is it distinct from your current practice?	Coherence
Does it have a clear purpose for patients and providers?	
Do you believe patients and providers will see the value and importance of e-PHR?	
Are the benefits likely to be valued by potential users?	
Do you believe it is right to engage in the use of the e-PHR*?*	Cognitive participation
Are the users likely to think it is a good idea?	
Will users be prepared to invest time, energy, and work into the use of the e-PHR?	
Do you think users can sustain involvement in the use of the system?	
Does e-PHR fit with existing skill sets and work practices?	Collective action
Will the system be supported and resourced?	
Do you think users will have confidence in the system?	
Will the e-PHR make people's work easier?	
What would you say about the likely effects on patients or healthcare providers and their work environment?	Reflexive monitoring
Are the effects likely to be perceived as advantageous for them?	
Will it be clear what effects the intervention has had once it has been in use for a while?	

The concurrently gathered qualitative and quantitative data were collected to thematic saturation. Once six participants' data were collected within a study group, the data were analyzed concurrently with the collection of data from each additional participant within a study group so that thematic saturation could become known and as such recruitment ended.

### Data Analysis

Demographic characteristics of the participants were described using Excel for simple descriptive statistics. NoMAD instrument and survey data were analyzed using R statistical software (30) for descriptive statistics including mean scores. Psychometric tests of the NoMAD instrument were conducted to examine the reliability and validity attributes of the instrument within the context of this study. Cronbach α testing was conducted on all four NPT mechanisms to measure the reliability or internal consistency of their constructs.

Interview data were transcribed and imported into Atlas.ti (31). Using coding as a heuristic discovery process (32), data were coded by the researcher (S.D.) for evidence of the constructs of NPT via a deductive qualitative approach. Concept coding was applied to the transcripts as the first cycle coding method. The second cycle coding method employed axial coding, essentially to identify dominant codes from the process of first cycle coding and to reorganize the data set such that the best representative codes were selected to form an emergent category. Lastly, comparing category to category and their related codes and data allowed themes to emerge. Throughout the complete analytic process, memo writing was used to capture the researcher's reflections on coding processes, code choices, any data that fell outside of the coding frame, and arising patterns in the data. Finally, emergent descriptive themes were identified, along with quotations of the participants that best illustrated the themes.

Consistent with the study design, the analyzed quantitative, and qualitative data were amalgamated to present results during the interpretation. That is, the analyzed data from the instrument, survey, and interviews were merged to a unified whole as a joint display framed by NPT for the purposes of complementarity in outcome interpretation and description.

## Results

### Participant Demographics

Twenty-seven participants in British Columbia, Canada, participated, including patients (*n* = 8), care providers (*n* = 11), and organizational providers (*n* = 8). The median age of patients was 20 years. Of the care providers, four were endocrinologists, two were family practice physicians, two were dietitians, and three were nurses. Of the eight organizational leaders who participated, six were government HIT leaders including chief medical information officers, chief information officers, and directors of information management/information technology (IT), whereas the remaining two were clinical directors. All considered themselves average or advanced in use of ITs. Characteristics of the participants are summarized in Table 2.

**Table 2:** Characteristics of study participants.

**Characteristic**		**Patients**	**Care providers**	**Organizational providers**
Age	Mean (years)	20.25	—	—
	Median (years)	20	—	—
Sex	Female	7	6	3
	Male	1	5	5
Geographic location	urban	5	6	7
	rural	3	5	1
Time in clinical practice (years)	1–4	—	2	—
	5–10	—	3	—
	11+	—	6	—
Working with EHR systems (years)	1–4	—	3	—
	5–10	—	5	3
	11+	—	3	5
Use of information and communications technologies	Advanced	6	2	5
	Average	2	9	3
	Basic	—	—	—
	Non-user	—	—	—

### Psychometrics of NPT-Based Measurement Instrument, NoMAD

Cronbach α test was used to measure the extent to which all constructs of an NPT mechanism measured the same concept. In this study, tests of internal consistency varied in terms of supporting the use of these items either as an overall measure of “normalization” (20 items, α = 0.60) or as four NPT mechanism measures (ranging from α = 0.33–0.80) (Table 3). Because of reliability issues identified in this study with the NoMAD instrument, the overall normalization score and the scores for two of the four NPT mechanisms, coherence and reflexive monitoring, were not used in further analysis as originally planned.

**Table 3:** Reliability of NPT mechanisms.

	**Coherence**	**Cognitive participation**	**Collective action**	**Reflexive monitoring**	**Overall normalization**
Cronbach α	0.33	0.8	0.8	0.55	0.60

### Normalization of e-PHR in Clinical Practice—An Integrated Summary

The integrated summary was arranged by NPT mechanism. The quantitative results offered descriptive statistics and indicated a direction of agreement with measures of normalization. The qualitative results used descriptive themes to provide a rich explanation of the findings using the participants' voices.

#### Coherence: Meaning and Sense-Making Work of Integrating e-PHR

Qualitative results indicated that e-PHR made sense as explained by two themes for the coherence mechanism: a *game-changing technology* and *sensibility of change*. Table 4 illustrates exemplar quotes by study group for each theme. Because of reliability issues identified in this study with the NoMAD instrument, the mean score for coherence was not used. To participants, e-PHR is a supportive approach to healthcare for patients and would formalize collaborative relationships, provide access to a comprehensive set of data, and offer timely and convenient communications. Participants made sense of e-PHR by noting its significant deviation from the current practice, requiring a shift in the culture of medicine and system policies, as well as a change in clinical workflow and business practices.

**Table 4:** Combined qualitative and quantitative results for coherence by study group.

**NPT**	**Descriptive themes**	**Exemplar quotes**			Mean score^*^± SD
		**Org providers**	**Care providers**	**Patients**	
Coherence	Converging views of meaning—a game-changing technology	“This is fundamental to where we need to go with healthcare. I see it as an enabling mechanism to put the ownership of a person's care more in their court, to shift the paradigm we have in our system from a didactic provider-dominated healthcare service to one that is truly person-centered”	“It challenges and pushes providers to be more patient-oriented and to have the conversation with patients about what is important to them vs. what's important to us as providers, which is often different”	“The shared decision making would kind of improve the relationship or make a deeper relationship between patients and their doctors. The technology is the conduit”	—
	Sensibility of change	“The important piece is, when a patient wishes to engage in this way and we have that option as a system to provide that [e-PHR] to them, there must be a shared understanding of what that means”	“Once we involve our patients, it is likely that we will have a better chance to have more compliant patients and better outcomes too, like less [disease] complications”	“So, I'll see the nurse and the dietitian and then my endocrinologist separately. I have to explain what's going on three different times before I can even start asking my questions. I think them having the whole story before I even go in would be really helpful”	

In general, while patients appeared less preoccupied with issues of this nature, care providers and organizational providers expressed openness to the required shifts in medical practice. In the words of OrgProvider1, “This is fundamental to where we need to go with healthcare. I see it as an enabling mechanism to put the ownership of a person's care more in their court, to shift the paradigm we have in our system from a didactic provider-dominated healthcare service to one that is truly patient-centered.” Participants understood the benefits of e-PHR as being a supportive approach for patients, an improvement in care efficiency, and a conceivable, positive impact on patient outcomes. The latter was most simply described by CareProvider3 as “Once we involve our patients, it is likely that we will have a better chance to have more compliant patients and better outcomes too, like less disease complications.” The ability for a care provider to have a more comprehensive set of patient health details prior to an encounter was seen overall by participants as very beneficial. Patient4 described it as “So, I'll see the nurse and the dietitian and then my endocrinologist separately. I have to explain what's going on three different times before I can even start asking my questions. I think them having the whole story before I even go in would be really helpful.” Shared access to patient health information and treatment strategies for integrated planning purposes was summed up by CareProvider4 as “When we don't know the details of what the other care providers on our team are doing, it makes it hard to make a cohesive shared plan.” Meaning was also linked to concerns of workload and workflow, and a limited shared understanding of purpose. CareProvider8 highlighted that “patients' time to engage with the system may not match the systems' time to engage with them.”

#### Cognitive Participation: Commitment and Engagement Work of Integrating e-PHR

There was very strong agreement by all participants across all four items related to the investment of commitment (overall mean score out of 5 was 4.6 ± 0.45) (Table 5). Participants felt they would be engaged with processes that promote participation individually and together. Interestingly, organizational providers' mean scores indicated the strongest levels of agreement across all items of this mechanism, revealing their assessment that care providers, and patients are up for the relational work needed to build and sustain a new practice around e-PHR. This was explained qualitatively by two themes: *sharing ownership of the work* and *enabling involvement*. Table 6 illustrates exemplar quotes by study group for each theme and mean score.

**Table 5:** Cognitive participation and collective action scores by study group.

	***N***	Cognitive participation score^*^ ± SD	Collective action score^*^ ± SD
Patients	8	4.4 ± 0.52	3.9 ± 0.33
Care providers	11	4.5 ± 0.39	3.4 ± 0.58
Organizational providers	8	4.9 ± 0.19	3.2 ± 0.44
Overall	27	4.6 ± 0.45	3.6 ± 0.53

* *1, strongly disagree; 5 = strongly agree*.

**Table 6:** Combined qualitative and quantitative results for cognitive participation by study group.

**NPT**	**Descriptive themes**	**Exemplar quotes**			Mean score^*^± SD
		**Org providers**	**Care providers**	**Patients**	
Cognitive participation	Sharing ownership of the work	“From a health system perspective, I have the least faith because our system doesn't do new initiatives very well. We don't put in the right supports. We don't put in the right governance. We don't put in the right funding. We have too many things that need to happen. There are too many conflicting priorities. There's politics which get in and redirect this to short term wins. This isn't a short-term thing”	“e-PHR is a great idea, but I'm less confident about how this could happen. Well I guess we would have to see how that would be, what kind of impacts it would be like, what the workflow is like, what kind of supports there are to understand it, introduce it and develop it”	“Helping diabetes management and strengthening the relationship between me and my healthcare provider. They have to be a little more involved in my life and I have to be clearer in my communications with them”	4.6 ± 0.45
	Enabling involvement	“If patients are feeling better supported and safer and their health is improving in a way that they notice, as opposed to indicators that don't really mean much to them, then I think the system will become self-perpetuating.”	“If the higher ups support e-PHR, they must provide protected time and the resources and the infrastructure that's needed” “I don't understand why you would stick to something when there's better opportunities”	“Anything that makes you feel like you are more on top of [disease management] and more in control is going to keep being used” “If you are seeing the benefit, then you would want to sustain it”	

* *1, strongly disagree; 5, strongly agree*.

To participants, e-PHR is the right direction for healthcare. Commitment to e-PHR was demonstrated through a shared interest in the collaborative, relationship-based focus of care. Patient7 explained that e-PHR would be “strengthening the relationship between me and my healthcare provider. They have to be a little more involved in my life, and I have to be clearer in my communications with them.” Participants expressed openness to new ways of working individually and together, as well as some fear of change and lack of systemic ownership of the change. Participants wanted to know that implementing e-PHR would have the right resources and supports in place to enable and sustain involvement. At the individual level, that involved upskilling such as education and training. At the clinic level, that required an examination of current processes for fit and the identification of additional resources and supports required to enable involvement. At the system level, the alignment of business drivers, such as the care provider funding model, was identified as fundamental. CareProvider7 highlighted, “In a fee-for-service environment, I am going to be relatively disinterested in this because I can't get paid for using it. In the value-based funding, I am going to be all over this because it allows me to maintain a high level of wellness in my population.” This was further demonstrated by CareProvider6 as “If the higher ups support e-PHR, they must provide the protected time and the resources and the infrastructure that's needed. The biggest issue with our healthcare system is these kinds of things become available, and they are implemented without any thought to the additional resources or training or time that is necessary to do that well.”

#### Collective Action: Effort Work of Integrating e-PHR

Weak agreement (overall mean score out of 5 was 3.6 ± 0.53) was observed with collective action processes requiring an investment in effort (Table 5). With some exceptions for patient participants, who had the highest level of agreement across all items of this mechanism, participants exhibited uncertainty about the work that operationalizes e-PHR. Both ambivalence and disagreement were observed in mean scores of organizational providers around a number of normalization processes that influence the mechanism of collective action, including (a) ease of incorporating the system into existing work, (b) disrupting working relationships, (c) confidence in other people's ability to use the system, (d) having sufficient resources available, and (e) adequate management support. Healthcare providers likewise neither agreed nor disagreed, as observed by their mean scores, about the ease of incorporating the system into existing work and about having the confidence in others' ability to use it, but their level of agreement with the other promoting processes of this mechanism was more positive.

This implementation mechanism was best explained qualitatively by one theme: *uncovering the challenge of building collective action*, and three subthemes: *assessing fit, adapting to change together*, and *investing in the change*. Table 7 illustrates exemplar quotes by study group for each theme and mean score. The effort to enact e-PHR would require an upskilling of care providers, a shared accountability among patient and care providers, and sufficient leadership and financial investment as part of the shift in the culture of medicine to patient-centered care. e-PHR exists in an environment of transparency and shared responsibility. OrgProvider2 explained that “We have more work to do across the system for sure around truly enacting a patient-centered approach to care. If the culture of care does not reflect the e-PHR, it won't be well-supported or used.” Participants agreed that patient accountability will increase, and overall a realignment of the care team and sharing of the various tasks will be required, including the task of care planning. OrgProvider3 noted that “the design of e-PHR might drive the redesign of the teams that provide care. It will put more onus on any member of the care team to establish the care plan in collaboration with the patient because ultimately it is putting more ownership back to the patient around their care plan.” Participants deemed that measuring and demonstrating benefit will foster an ongoing investment. If population-level improvements are demonstrated, the system will resource e-PHR. CareProvider3 shared that “seeing benefits and return on investment from the collaborative decision-making with their patients will bring the support.”

**Table 7:** Combined qualitative and quantitative results for collective action by study group.

**NPT**	**Descriptive themes**	**Descriptive subthemes**	**Exemplar quotes**			Mean score^*^± SD
			**Org providers**	**Care providers**	**Patients**	
Collective action	Uncovering the challenges of building collective action	Assessing fit	“We have more work to do across the system around truly enacting a person-centered approach to care. If the culture of care does not reflect the SDM via PHR, it won't be well supported or used” “I don't believe we've done a good job of really digging deep into what [is required] to change the culture of care, really create and develop the skill set around that”	“We will be uncovering people with poor skills on doing the engagement that should have been happening all along without the tool. The tool will expose gaps that in turn might precipitate more anxieties on the part of the providers, because they are going to be asked to do stuff that we took for granted they should be doing all along”	“For young people, we grew up with technology, so for us it is a second language.” “I don't think it would be that much of a change in effort [for patients]; we don't get to not be thinking about [our disease]. So, I think the only difference with this new technology is that it would be shared”	3.6 ± 0.53
		Investing in the change	“e-PHR is likely to be sponsored in theory but not resourced to the extent it needs to be”	“Seeing benefits and return on investment from those conversations and the collaborative decision making with their patients will bring the support”	“If we introduce the concept [to patients of disease management] being in your hands, not just your doctors at an earlier age that would be beneficial”	
		Adapting to change together	“We need to clarify the road map and to establish some amount of centralized control, but we don't want to stifle creativity. That is the complexity and the art of public policy”	“Patients automatically assume that you are checking lab work for them daily. But I don't necessarily have the time to be going through and making sure their HbA_1c_ is in target. So, I think figuring out where is the ownership?”	“It would be relinquishing some of the accountability from the providers' side over to the patient which they have not typically been accustomed to… and the different practitioners and teams, it would be moving everybody up I think to a more parallel playing field around roles and responsibilities”	

* *1, strongly disagree; 5, strongly agree*.

Participants expressed an ambiguity toward the ability to adapt to change together; i.e., e-PHR may not easily integrate into existing work without a disruption to current relationships and processes and some lack of trust and confidence in others' ability to carry out tasks required to enact e-PHR. Shifting roles toward partnership was explained by Patient2 as “it would be relinquishing some of the accountability from the providers' side over to the patient which they have not typically been accustomed to… and the different practitioners and teams, it would be moving everybody up I think to a more parallel playing field around roles and responsibilities.” In adapting to the change together and to satisfy the practical process issues of integrating this new paradigm into clinical practice, participants identified the importance of a top-down strategy and policies with embedded practical experience from on-the-ground clinical practice, yet there is an uncertainty about how well they will merge. Participants argued that the time is ripe to really get it right, citing the opportunities for efficiencies in the care approach are well-worth the realignment of workflow and business practice, but there was a lack of clarity around what those new business rules might be. CareProvider7 shared “Patients automatically assume that you are checking lab work… daily. But I don't have the time… So, figuring out where is the ownership?” Participants expressed that expectations need to be established that are respectful of care providers' workload yet drive patient engagement. CareProvider6 stated, “one concern I have is the pace of information transfer today and the expectation of response… Would that be disengaging for a patient to reach out and then have nobody answer until Monday morning?”

#### Reflexive Monitoring: Appraisal Work of Integrating e-PHR

Participants appraised e-PHR as explained by two themes for reflexive monitoring: *reflecting on value* and *monitoring and adapting*. Table 8 illustrates exemplar quotes by study group for each theme. Because of reliability issues identified in this study with the NoMAD instrument, the mean score for reflexive monitoring was not used. To participants, e-PHR would nurture engagement and collaboration by removing barriers to care and increasing care efficiency and effectiveness, but outcomes must be measured and benefits demonstrated. CareProvider11 assessed the shift in the care approach as valuable because “by engaging patients in their care this way and providing them with this kind of empowerment and increasing frequency of contact, that would translate into better outcomes.” Patients shared how the timeliness of connections would improve their experience of care. Patient8 described it in the following manner, “Instead of it being one little issue that turns to a big issue, you can fix them with your doctor as they show up.” Participants perceived improvement in the effectiveness of care—specifically, the enabling of collaboration with an ease to communications and a more comprehensive picture of the patient's health. Participants argued that the integration of the care team online with access to a more complete patient profile and various communication mechanisms supports the patient in the day-to-day management of health. Patient6 offered, “Communication would be better and therefore probably be less risks at home because you'd be able to share information about the problem and then solve the problem faster instead of being like ‘oh well my appointment is in 2 months so I'm going to fix it then.’” Getting clear about the effects of e-PHR came with an imperative from participants to measure and demonstrate outcomes on an ongoing basis and to monitor workload and adapt clinical practice accordingly. OrgProvider7 explained that the effects will only be evident if “we are purposeful about bringing that forward and measuring it.”

**Table 8:** Combined qualitative and quantitative results for reflexive monitoring by study group.

**NPT**	**Descriptive themes**	**Exemplar quotes**			Mean score^*^± SD
		**Org providers**	**Care providers**	**Patients**	
Reflexive monitoring	Reflecting on value	“A more respectful engagement in care. Being more of a partner to [healthcare] service, being more respectful of the values and beliefs and recipients of the care”	“By engaging patients in their care this way and providing them with this kind of empowerment and increasing frequency of contact, that would translate into better outcomes”	“Instead of it being one little issue that turns to a big issue, you can kind of fight all these little battles at once and fix them with your doctor as they show up”	—
	Monitoring and adapting	“The effects will only be evident if we are purposeful about bringing that forward and measuring it”	“Patients share some results and ask me what I should do. How does that get remunerated? Do I have to do a billing interaction every time I have a 10-s interaction with the portal? …Those kind of implementation details will make a big difference”	“Communication would be better and therefore probably be less risks at home because you'd be able to share information about the problem and then solve the problem faster”	

* *1, strongly disagree; 5, strongly agree*.

#### Bridging Theme of Integrating e-PHR

One qualitative theme resulted that spanned all NPT mechanisms. This theme, *Really get it right!—United views of system usability, intelligence, and connectedness*, may be indicative that all four cognitive and behavioral processes that influence the integration of e-PHR into clinical practice are impacted by aspects of its ultimate design, which remain elusive to participants in this early design/preimplementation phase of e-PHR. Because these repeating ideas could not be connected to any one NPT mechanism, it was described and added to the integrated results as a bridging theme. This theme captured the repeating idea of really getting this technological innovation correct in terms of a usable, intelligent, and mobile design within a standard-based, federated, technical infrastructure.

When describing what would keep people motivated to continue taking part, the topic of usability of e-PHR was associated with an alignment to other current, intuitive, and acceptable ways of working, as argued by CareProvider2, “I can't stress enough that the program has to be user friendly in order for it to be accepted easily. I think that user-friendly would be that intuitive piece. You know, if you pick up an iPhone, it's quite intuitive, but if you picked up a different model, you really have to struggle your way along.” Designing e-PHR with patient mobile access in mind was expressed by participants throughout. Patient4 explained when speaking about what makes the effects of e-PHR seem beneficial: “If nothing else, while we are moving around [geographically], you know that you are at least still connected to people that care about your health.” The importance of system intelligence for e-PHR as it relates to managing and presenting the data and information and adaptive decision support was also highlighted across all normalizing mechanisms. Patients often and easily alluded to their need for a simple yet comprehensive dashboard to manage their health. The overall management of data and information by the system in terms of a usable presentation style and search functionality without increasing workload was often identified by care and organizational providers. As one example, CareProvider3 described how the value of e-PHR is judged, “I would just hope that with all the latest technology that's available they would be able to have it organized in a way that's easily searchable. So, if you were looking for their kidney function, you wouldn't have to traipse across all of the files.” The significance of an intelligent system for a computer-tailored approach to clinical decision-support was described by some care and organizational providers. CareProvider4 stated, “Knowing all the decision support intelligence stuff, I think it would really help a lot of patients' needs to not even have to reach out to a care provider if some of that information was more readily in their hands with some intelligent decision support behind it—alerts, reminders, those types of things.” Finally, the notion of an integrated ecosystem of EHR systems cannot be underestimated as participants often and, across all normalization mechanisms, described its relevance and importance. As one example, when speaking about what makes the effects of e-PHR seem beneficial, OrgProvider4 shared, “We need a standard-based infrastructure that these things can plug into. This environment gives you a space where multiple vendors can create new products, start-ups that can plug in; they don't have to build the entire stack, they can just build what they are specializing in and interact with the rest of the system.”

#### Practice-Related Outcomes of Integrating e-PHR

Participants strongly agreed that e-PHR would positively affect engagement in self-management decision-making and agreed that it would become a normal part of work. The potential practice-related outcomes generated two descriptive themes: care is efficient, and care is patient-centered. The mean scores for the practice-related outcomes by study group and overall were calculated (Table 9). Table 10 illustrates exemplar quotes by study group for each theme and mean scores. The practice-related outcome of normalization obtained general agreement (mean score = 3.9/5); however, the organizational providers expressed more ambivalence to this potential outcome (mean score = 3.2/5) compared to care providers (mean score = 4.1/5) and patients (mean score = 4.0/5). This outcome was described by participants as only likely if the required shift in the culture of medicine toward patient-centered, team-based care occurs. As OrgProvider2 noted, “the care approach has to marry and reflect that same philosophy and culture. If those two things are in place, I believe it will positively impact engagement.”

**Table 9:** Mean practice-related outcomes scores by study group.

**Practice-related outcome**	***N***	Patients mean score^*^ ± SD	***N***	Care providers mean score^*^ ± SD	***N***	Organizational providers mean score^*^ ± SD	***N***	Overall mean score^*^ ± SD
Positively impact engagement in self-management decision-making	8	4.4 ± 0.74	11	4.5 ± 0.52	8	4.6 ± 0.52	27	4.5 ± 0.58
Easier to participate in SDM	8	4.8 ± 0.46	11	4.4 ± 0.50	8	4.8 ± 0.46	27	4.6 ± 0.50
Become a normal part of my work	8	4.0 ± 0.53	11	4.1 ± 0.54	4	3.2 ± 0.50	23	3.9 ± 0.60
					4	Not relevant to my role		
Easier to support patients in self-management	8	4.4 ± 0.52	11	4.3 ± 0.65	8	4.8 ± 0.46	27	4.4 ± 0.58

* *1, strongly disagree; 5, strongly agree*.

**Table 10:** Combined qualitative and quantitative results for practice-related outcomes by study group.

**Practice-related outcome**	Survey mean score^*^ ± SD	**Descriptive themes**	**Exemplar quotes**		
			**Org providers**	**Care providers**	**Patients**
Positively impact engagement in self-management decision making	4.5 ± 0.58	Care is efficient Care is person-centered	“The care approach has to marry and reflect that same philosophy and culture” “It feels to me that there is a cultural elitism thing there, which needs to go away. So, this kind of tool would help with that because it would drive the culture toward partnership” “The magnitude of impact on hard clinical outcomes is probably going to be low. I think if we don't focus on that and be a little more holistic in our health approach and think does this improve treatment satisfaction or does it reduce diabetes distress scores or quality of life score, I think it probably will be positive”	“We are actually able to directly contact [our patients] and have a conversation without them having to come into the clinic” “It gives information to the patient that they've never had… and shifts the relationship to more of a collaborative one” “it just makes it way easier for those patients to access care… and they don't have to be in town” “[patients] might go home and think, what kind of instructions did [my provider] give me again and if it was all in e-PHR, then I think would make it easier for patients and care providers too” “If we are truly doing person-centered care, around their beliefs and values, we might find that some of the things we know clinically a person should be doing or moving toward may not be the care plan for that individual”	“Rather than just thinking of [our concerns], we'd actually be acting on them” “It makes you feel more involved, like you have more of a voice in your own health, as weird as that sounds” “I think a lot of factors get left out such as stress levels and activity levels if you have been traveling, for example, or you changed your diet… if [care providers] could just see kind of what you see every day, with your activity changes and emotional changes, it might be a little easier to fine tune how to care for yourself if they had that extra information that usually gets lost”
Become a normal part of my work	3.9 ± 0.60				
Easier to participate in SDM	4.6 ± 0.50				
Easier to support patients in self-management	4.4 ± 0.58				
Reduce diabetes complications	3.6 ± 0.58				

* *1, strongly disagree, 5, strongly agree*.

There was strong agreement by all participants that e-PHR would positively affect engagement in self-management decision-making (mean score = 4.5/5), with 14 strongly agreeing, 12 agreeing, and one participant neutral. Participants perceived an increased efficiency of care and everyone being more informed. The efficiency of care appeared to arise from a level of convenience that is both desired and perceived as available with e-PHR in terms of access to and the provision of care. As Patient8 described, “If we had this system at our disposal to use and vocalize some of the concerns we have, rather than just thinking of them, we'd actually be acting on them, so I think it would have positive effects on being engaged in your own care.” In terms of convenient access to care, CareProvider9 shared, “It just makes it way easier for patients to access care… and they don't have to be in town. They can be in Vancouver. They can be in Montreal. They can be wherever they want to be and still stay connected to their clinic.”

According to the overall mean scores, all participants perceived e-PHR would make it easier to participate in SDM (mean score = 4.6/5) and to support patients in managing their own care (mean score = 4.4/5). From the patient perspective, it appeared as though access to information would empower them to participate in decision-making as expressed by Patient7: “It makes you feel more involved, like you have more of a voice in your own health, as weird as that sounds.” Participants described that having access to the “big picture” would allow patients to take more ownership of their own care and related decision-making. CareProvider7 noted that the ability to participate is because “it gives information to the patient that they've never had, which may empower them to engage more frequently and shifts the relationship of care provider and patient to more of a collaborative one.” When it comes to having access to comprehensive information, such as a care plan, outside of the care encounter, CareProvider3 pointed out how helpful this would be: “[the patient] might go home and think, what kind of instructions did they give me again, and it would be all in e-PHR.” Participants described the ability to support patients in their care as a result of treatment decisions being made that will more likely be followed because they are made with the patient, taking into account the whole person with a more comprehensive set of data. Patient1 shared how having a more complete understanding of the person would aid decisions and enable ensuing actions to be more accurate. She shared, “I think a lot of factors get left out such as stress levels and activity levels if you have been traveling, for example, or you changed your diet. A lot of those tiny factors have a really big effect on your managing your health, and I think those can definitely be missed in appointments when [care providers] are just looking at the [laboratory] numbers… if they could just see kind of what you see every day, with your activity changes and emotional changes, it might be a little easier to fine tune care if they had that extra information that usually gets lost.”

#### Overall Interpretation for the Normalization of e-PHR

**Figure 3:**
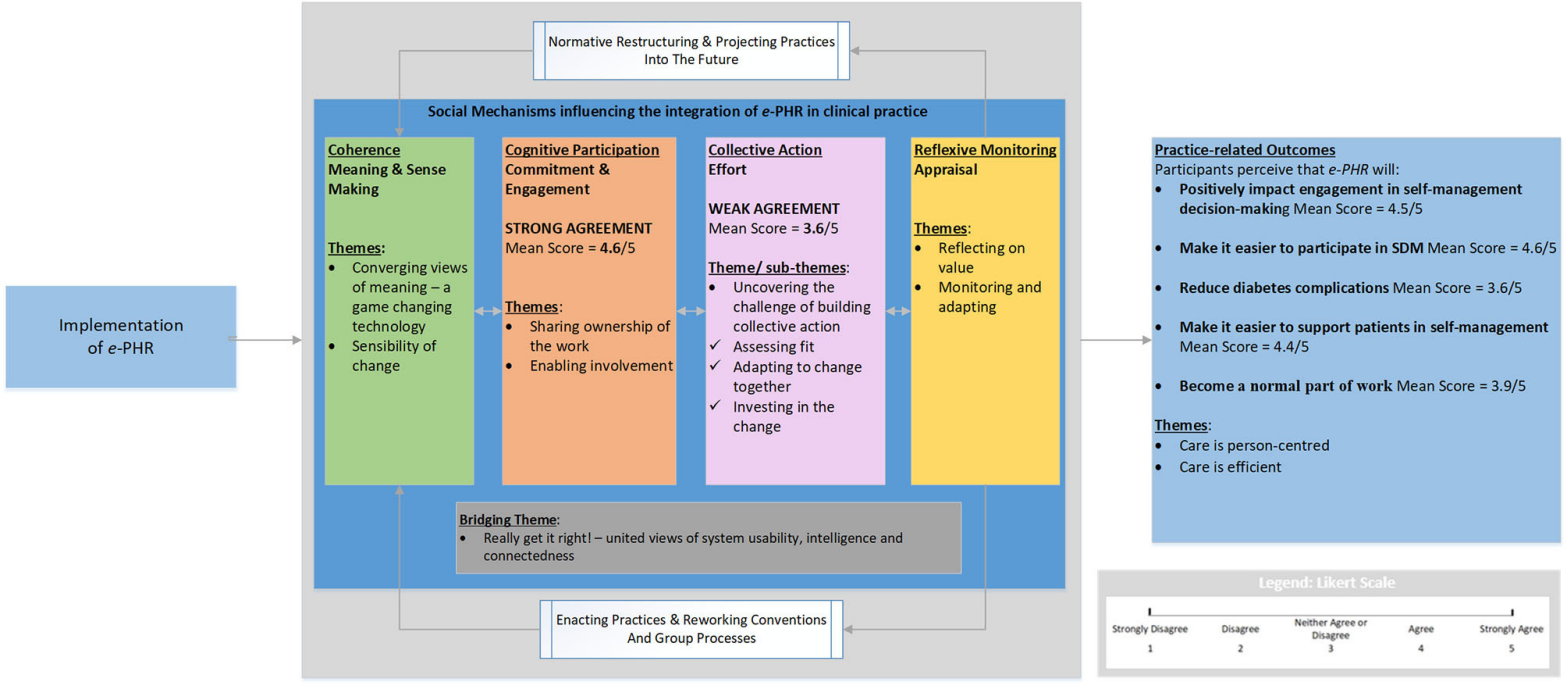
Overall interpretation for the normalization of e-PHR.

Overall, participants' cognitive and behavioral processes of sense-making, commitment, and appraisal to normalize e-PHR in practice to engage patients in self-management decision-making appeared encouraging. However, the collective action mechanism or implementation effort required to enact and sustain e-PHR was less positive, as indicated by the lower mean score and the description of the concepts of the theme and subthemes. The mean scores of the two NPT mechanisms, cognitive participation and collective action, and their qualitative themes corroborated each other. Figure 3 illustrates the overall results in response to the research questions, using a joint display of mean NPT mechanisms scores and themes benefitting from the NPT framework and the mean scores and themes for the practice-related outcomes.

## Discussion

The discussion section examines (a) the psychometric tests of the NoMAD instrument in this study, (b) the implementation work of e-PHR and its potential to integrate into clinical practice in terms of the four NPT mechanisms, and (c) the potential practice-related outcomes.

### NPT-Based Measurement Instrument, NoMAD

The NoMAD tool was chosen as it is the first quantitative measure based on NPT. In the test for reliability, both the cognitive participation and collective action mechanisms in this study had strong internal consistency, but the internal consistency of the coherence and reflexive monitoring mechanisms were weak. The overall measure of normalization had a Cronbach α of 0.60 and may be explainable by the poor reliability in two of the four NPT mechanisms. The small sample size may be a significant factor in this study. Modification of the NoMAD instrument and further primary studies examining its psychometric performance are needed before it can confidently be used as a reliable measurement instrument of NPT. Notably in this research, there was congruency between the NoMAD scores for cognitive participation and collective action and the qualitative data. This result strengthened the study and gave depth to the findings by providing a fuller understanding of these two normalization processes.

### e-PHR: Implementation Success Evaluated

Coherence refers to peoples' understanding of an intervention and the sense-making work involved in establishing this understanding. Participants made sense of e-PHR by noting its deviation from the current practice. Participants concurred that the transparency and fluidity of data associated with e-PHR and the processes that e-PHR avails users differ significantly from the way care is carried out today. For patients, access to comprehensive and timely data and the responsibility and power for decision-making offer opportunities to effectively self-manage and communicate confidently with providers (33).

While e-PHR made sense to participants, its deviation from current practice will require numerous shifts at the individual, organizational, and system levels. In-line with Scholl et al. (34), these shifts include the following: align clinical workflow and payment models, foster a shared understanding, and create supportive legislation and policies. At the individual level, this relates to knowledge and skills, workflow within care team, perceived influence, change in patient–provider accountability, and loss of control. Miles and Asbridge (35) articulate valuable methods to move from the current didactic provider-dominated service through the rhetoric of patient-centered healthcare to implementation and outcomes, including mapping deficiencies and deficits and upskilling providers. The literature has described the value of shared accountability with interprofessional care teams in terms of clarity of roles, tasks, and goals (36), but with the emergence of the patient as partner in digital care, a new shared model of accountability is needed.

At the organizational and system level, shifts were conveyed as changes in the culture of medicine and changes in health system processes and policies. To enable a shared digital-health information ecosystem, changes in health-system policies require review in terms of provider incentive models and privacy legislation. There is a growing body of evidence about the potential effectiveness of provider incentive models that align payment with quality performance (37) and drive adoption of a shared digital-health information environment (38). Further, and in alignment with the work of Brennan et al. (39), this research illustrated the need for system-wide efforts to involve patients in the design of technological solutions such as PHRs, whereby patients will invest in meaning, commitment, and effort because the design is grounded in their needs and preferences.

Participants saw the sensibility of e-PHR through its expected benefits and how it affects the work of patients and care providers. Access, connectedness, and convenience were seen by participants as the most supportive aspects of e-PHR for patients. Linking access and ease and timeliness of communications to measured outcomes will favorably support ongoing sense-making work of e-PHR. Patients explained that with e-PHR use they would feel more supported because decisions need to be made often and, for example, not only at a prescheduled 6-month follow-up appointment. In a study on designing a patient portal for patient-centered care, patients identified the importance of decision-making with their provider and wanted to be able to view the evolution of their health over time and to be notified when health changes were identified (40). Patients also described a likely improvement in their experience of care by increasing their confidence in their ability to self-manage. In fact, improved quality of healthcare through improved access to and sharing of information and improved ability of patients to manage their own healthcare were the identified patients' experiences in a study exploring their perceptions and experiences with PHR use (33). The authors (33) also conveyed that maximum benefits would be realized when PHRs contain a complete collection of relevant health information. Given the interconnected design of e-PHR, it is expected that optimal care quality will be attainable.

For care providers, the sensibility of e-PHR is overshadowed by concerns related to their ability to operationalize workflow and the anticipation of an increased workload. In a study relating new responsibilities with PHR use, Hill et al. (41) found intersecting concerns with PHR use on time demands and liability within already heavy workloads of care providers. The sheer volume and fluidity of data with e-PHR highlight issues of alert workload, a well-known problem identified by care providers that is still debated (42). Research on the topic of EHR alerts and patient safety has illustrated that patient safety is at risk with increased inappropriate firing of alerts, which has led to alert fatigue and the potential for ignoring important notifications (43). More investigations are needed regarding the benefits and impacts of e-PHR use to better understand, mitigate, and support changes to care processes and policies.

Organizational providers expressed ambiguity around the patients and care providers having a shared understanding of the purpose of e-PHR. This may be an emergent property of implementation. That is, over time, a coherent and shared understanding will likely develop as patients, care providers, and organizational leadership become more familiar with the practice. Still, in another implementation study using NPT (25), lack of agreement over the intervention's purpose was observed, and the authors indicated that clarifying its purpose at the outset would likely remove resistance by reducing the extra work caused by uncertainty. Thus, to successfully root e-PHR into practice, its purpose must be well-defined, common, and made to be intrinsic to healthcare. Additional work is required to foster this shared understanding as well as to manage the expectations of both patients and care providers around care services as they shift with the introduction of this game-changing technology.

Cognitive participation is the relational work that people do to engage and commit to a new intervention. Participants felt strongly that they would engage with processes that promote participation individually and together. This insight requires explicit attention of implementers such that strengthening of patients' and care providers' sense of their collective experience will translate to commitment and engagement in the practice of SDM via PHR.

While participants are committed to e-PHR, strategies are needed to address the collective resistance to change and the fear of non-systemic ownership of the change, as well as to manage patients' and care providers' expectations and allocate substantive resources and training. Thoughtful change management efforts, such as training and resources to support the change, will be imperative to maintain this openness to new ways of working and shift any fear and resistance. Training is one way of communicating what is involved and the possible benefits. Indeed, training led to high levels of involvement and commitment in one implementation study underpinned by NPT where this preparatory exercise led key people to drive the intervention forward and get others involved (22).

When describing shared ownership of the work, participants identified e-PHR as the right direction for healthcare with its increased levels of engagement, collaboration, and practice efficiencies. However, participants emphasized that the ownership is not only by patients and care providers, but also by organizations and the healthcare system. Active leadership has been identified as crucial to the implementation of new practices and especially effective when focused on the redesign of supportive policies and organizational structures (44). A common and significant concern identified by participants was that an inadequate level of system leadership and resources could hamper the success of e-PHR. Resources and funding are not new barriers to the implementation of EHRs. In fact, a systematic review of users' perspectives with EHR implementation indicated that 19 of its 52 studies considered the lack of funding as a barrier to implementation (45). A well-resourced financial outlay for sustainment was also described by participants in their appraisal work and effort required to enact e-PHR. Strategies and operational solutions to manage this barrier are paramount.

Collective action is the operational work that people do to enact an intervention. In general, the collective action aspects of the work required to enact e-PHR appear low and point to a set of inhibiting factors on which to focus future efforts. In other studies using NPT, this mechanism assisted in identifying the factors to optimize the intervention for testing in a larger-scale trial or for a subsequent full-scale implementation (46, 47). The effort to enact e-PHR will require an upskilling of care providers, the redesign of teams and processes, a well-resourced investment, and support to bolster a systemic willingness to adapt to change together.

Participants in this study exposed an underexamined issue related to care providers' skills (or lack of) to engage the patient in decision-making and care. A study on physician SDM skill acquisition confirmed that additional skills are needed and should be delivered through medical education (48) and continuing education programs (49). Canadians' vision for the creation of better health through digital solutions is to establish conditions for greater patient involvement in and increased transparency of decision-making (50). Enhancing care providers skills around the use of SDM and collaborating in teams with the patient as a partner in digital care will likely be invaluable to the collective action work of implementing e-PHR. Related is a study where the use of EHR was interpreted as a possible threat to professional autonomy of physicians (45). The need to support providers with the transition of their care practices to an environment of shared responsibility and transparency is imperative. If not addressed, it could have a significant negative impact on both the perceived usefulness of e-PHR and the willingness to invest effort into operationalizing it.

Most participants expressed doubt about the likelihood of a well-resourced implementation explaining that, while e-PHR was likely to be sponsored in theory, it was not likely to be resourced to the extent needed; historically, this has been the case with other healthcare interventions. A lack of IT infrastructure and resources that could adequately support an intervention were identified as impeding factors of collective action in other implementation studies (22, 25). Demonstrating incremental benefits is likely to drive effort, and it turn these advantages should drive investment.

Participants expressed ambiguity regarding their ability to adapt to change together; i.e., e-PHR may not easily integrate into current practice without a disruption to relationships and processes. Trust and collaborative partnerships for optimal care play an important role here, especially given the perceived relinquishing of control and accountability toward the patient. In a study to understand how patient privacy concerns affect their disclosure of health information, the authors (51) found the perception of high-quality care reduced the likelihood of withholding information and may be an effective strategy to foster patient–provider trust. Further investigations around trust and relationships between patients and care providers may uncover strategies for them to collectively adapt. Contrary to participants' concern of a disruption to relationships and processes, use of PHRs can strengthen patient–provider relationships (52).

In this study, the cognitive and behavioral processes of meaning, commitment, and engagement did not translate strongly to enacting collective implementation efforts. The lack of translation from one mechanism to another was consistent with finding of Burau et al. (44), where the authors found that participants identified a health promotion intervention as meaningful, yet it did not translate into an engaged, collective implementation effort. NPT developers have acknowledged that the dynamic and contingent activities of the four mechanisms and their production and reproduction evolve over time (53). Future research should pay closer attention to the complex interplay between the four NPT mechanisms; for example, how the intervention, the context, and the individuals determine how meaning and engagement are translated into enactment.

Reflexive monitoring refers to how people evaluate an intervention, the collection, and use of feedback and how the intervention changes over time. Given that this evaluation was carried out at the design and planning stage of e-PHR with no tangible solution for participants to assess, the value of this reflective process may not be relevant. The process of reflexive monitoring is likely better suited to implementation stages that are further along than planning. That said, participants in this study did appraise the value of e-PHR as having the potential to nurture engagement and collaboration by removing barriers to care and improving patient care experience.

Participants conveyed an imperative to measure and demonstrate outcomes on an ongoing basis and adapt at the system- and practice-level accordingly. Similar to results reported by Dickinson et al. (22), participants were interested in more formal evaluations of the intervention and how positive effects could be maintained beyond the defined implementation period. In another study by Yeung et al. (54), the reflexive monitoring mechanism offered constructive insights by care providers regarding implementation of a screening intervention; specifically, it found providing quarterly feedback reports gave providers an opportunity to reflect and appraise their work and identify changes within their control that could be made to their practices to facilitate screening. These insights are valuable and should be utilized in a future implementation of e-PHR.

Healthcare system decision makers need to take strategic and operational leadership on technological infrastructure to center the patient in care and engage them within an integrated EHR ecosystem using the patient-facing version, the PHR, which is where their health information for SDM lives.

### e-PHR: Potential Practice-Related Outcomes

All participants perceived e-PHR as a technology to both engage the patient and make it easy for the patient to participate as a partner in their care and decision-making in a manner that is respectful of their care preferences. PHRs have been identified as tools to improve patient engagement (55–57), particularly in engagement related to self-management (58). Any future implementation of e-PHR should evaluate measures of patient engagement and SDM.

Weak agreement was observed among participants around whether e-PHR would become a normal part of their work. Organizational providers indicated the greatest uncertainty. This may signal a greater awareness on their part, relative to patients or care providers, of the breadth and depth of organizational- and system-level challenges required to integrate e-PHR into clinical practice. In a scoping review of 48 articles on organizational- and system-level characteristics that influence the implementation of SDM, Scholl et al. (34) categorized the influencing organizational characteristics as (a) leadership, (b) culture, (c) teams, (d) priorities, (e) workflows, and (f) resources, and the influencing system-level characteristics as (a) incentives, (b) policies, (c) culture, and (d) education and training. The authors (34) argue that tailoring strategies to address these influencing characteristics could improve implementation success. Given that many of these characteristics were identified in this study, a future implementation of e-PHR would be well-served by distinguishing which levels of organizational leadership should take action to address a specific influencing characteristic, for example, which leadership level should set related priorities and resources, support multidisciplinary patient–provider teams, and disseminate strategies to support patient and provider workflows. Further, the healthcare system and their organizations should be methodical and unified in their approach to create a culture that supports SDM via PHR.

### Strengths and Limitations

The application of mixed-methods gave depth to the descriptive study with qualitative results corroborating with quantitative results using NPT measurement tool. Further, thematic saturation within study groups was achieved adding strength to our findings. Although a small sample size, the sample offered the research the desired maximum variance of multiple study populations in British Columbia with regard to several key characteristics, such as sex, geographic location, and number of years in clinical practice as shown in Table 2, to best understand the topic while reaching a reasonable saturation in the collection of data. In other descriptive studies with similar approaches, data saturation was reached within similar range of sample size (20, 25). While the use of a non-representative sample does not permit generalizability to other populations, the high “information power” (59) of the participants with the specific clinical condition of diabetes is in line with the vision for digital solutions of Canadian citizens with varying clinical conditions (50) and adds both credibility and transferability of the results and makes possible the drawing of valid conclusions.

In terms of the quantitative results from the NoMAD instrument, caution in interpretation of the results is necessary given the large number of statistical tests performed relative to the small sample size. A strength of this study was its mixed-methods approach. That is, the outcomes of the NoMAD instrument for two of the four NPT mechanisms were consistent with the qualitative data. In a recent mixed-methods study using NoMAD (24), the NoMAD instrument outcomes were consistent across all four NPT mechanisms with qualitative data, although no psychometric testing of the instrument was completed.

In terms of the qualitative data analysis, only one researcher coded the data. Every effort was made by the researcher to remain open to the possibility that data may fall outside of the NPT coding frame used in this research and therefore required further examination to determine if important concepts or ideas were being missed.

It is possible that study participants were particularly interested in technology (such as characteristics known to early adopters) or the advancement in diabetes care. Their views of e-PHR may not reflect the views of people with other health conditions or interest in the use of technology. Additional research across other clinical domains is needed.

Finally, this study was a preimplementation assessment, and as such, there was no tangible solution for participants to assess; rather, they did so theoretically. Additional feasibility and usability studies with a developed system would be valuable for results to be grounded in the participants' experience with use of e-PHR.

### e-PHR: Implications for Successful Integration

This study identifies key aspects for future development, implementation success, and usage of e-PHR.

First, the need to consider user perspectives in the development and deployment of HITs has been established both in academic research forums and in public discussions. In this work, the cognitive and behavioral processes associated with e-PHR implementation success were examined from the perspectives of patients, care providers, and system-level leadership. User-involved approaches increase the likelihood of implementation success because they are aligned with the needs of the users (26). As such, the findings in this research indicate high practical relevance. For system developers, an advanced prototype may undergo usability testing to ensure that an implementation of the system does not fall short of expectations of its users. In line with this research approach, every effort should be made by developers and implementers to put in place processes for ongoing engagements with users throughout the implementation stages to both inform and educate them and be informed and guided by them.

With the integration of e-PHR into the digital health ecosystem, patients and care providers will have enabling processes, tools, and technologies in place for SDM and access to health information and communications that align with the ways of working today. These enablers create opportunities for more engaged patients and better health outcomes; nevertheless, care providers' workload, clinical team workflow, and medical–legal issues require further investigation. Further, system implementers and organizational leaders can apply the learnings from this preimplementation evaluation of e-PHR with a focus on boosting enablers and bridging the barriers for a successful future implementation.

For the healthcare system, examination of policies, incentives for care providers, and operational and strategic pathways to resource and advance the required technological infrastructure of connected systems are needed. As we move into an ecosystem of connected care, information sharing across private and public domains is required, and current policies and governance structures must align. Considerations will be needed to encourage system developers to reengineer their products (or new vendors to design products) to align with the functional requirements of SDM via PHR and identified standards and protocols for seamless information exchange. With a change in the way care providers are remunerated, the foundation will be laid for new ways to engage patients and support their care. This will require additional education and training for care providers and patients on SDM and around office efficiency within connected care systems.

Finally, the healthcare system and its organizations should be methodical and unified in their approach to shift a didactic provider-dominated medicine culture to align with a patient-centered philosophy that supports e-PHR and a quality improvement spirit, including mapping deficiencies and deficits, measuring outcomes with e-PHR use, and highlighting excellence.

### Concluding Remarks

PHR technology designed to enable SDM and built on an interconnected architecture can offer a complete, shared, and balanced profile of the patient and provision of personalized decision support and communications tools. This preimplementation process evaluation, grounded in NPT, was extremely valuable for informing future implementation of e-PHR, including perceived benefits and barriers. The use of NPT in planning stages of implementation projects provides a real-world context in which to explore the work that will take place to integrate a new practice or technology and important data to redirect or stop planning if the likelihood of normalization is low (60).

The results of this study indicate that NPT offers an applicable framework in which to detail the processes known to influence successful integration of HITs into their complex sociotechnical healthcare environment. In detailing the use of NPT, it is, in and of itself, a valuable contribution to implementation science theory (60). In addition to the usefulness of NPT in the preimplementation stage, its use should be considered at all stages of the system design life cycle for e-PHR. For example, with an e-PHR prototype developed and deployed in small scale, the processes routinely operationalized in everyday work by care providers and patients could be evaluated for optimization prior to deploying in a larger scale.

The state of SDM in clinical practice is not a question of whether we should do it or not; rather, it is a question of successfully integrating the practice of SDM for patients and care providers within today's evolving EHR–PHR ecosystem and patient-centered care approach, and tomorrow's interconnected, mobile, and ubiquitous technology environment. Using the NPT framework, findings from this preimplementation process evaluation indicated participants invest in sense-making, commitment, and appraisal work of this PHR designed to enable SDM. However, integration of e-PHR into normal clinical practice is not quite ready for prime time and will only be attained when systemic effort is invested to enact it. Further research is needed to explore this gap to inform priorities and approaches for future implementation success.

## Data Availability Statement

All datasets generated for this study are included in the article/Supplementary Material.

## Ethics Statement

The studies involving human participants were reviewed and approved by University of Victoria Harmonized Human Research Ethics Board. The patients/participants provided their written informed consent to participate in this study.

## Author Contributions

SD led the research and was responsible for the literature review, knowledge modeling, data collection and analysis, interpretation of the data, initial drafting of the manuscript, edits of the final manuscript, and approved the final manuscript.

## Conflict of Interest

The author declares that the research was conducted in the absence of any commercial or financial relationships that could be construed as a potential conflict of interest.

## Supplementary Material

Supplementary Material 1 User-validated functional model for e-PHR.

Supplementary Material 2 The NoMAD: a measurement instrument of NPT.

Supplementary Material 3 Survey of practice-related outcomes.

Supplementary Material 4 Semistructured interview guide.
