# Supplementary material for: Ready for Prime Time? Using Normalization Process Theory to Evaluate Implementation Success of Personal Health Records Designed for Decision Making
Source: Front Digit Health. 2020 Nov 20;2:575951. doi: 10.3389/fdgth.2020.575951 (PMC8521962; doi:10.3389/fdgth.2020.575951)
Supplement: Supplementary Material 1 — User-validated functional model for e-PHR. [file Data_Sheet_1.PDF]

Supplementary Material 1: NoMAD Measurement Instrument

This NoMAD Measurement Instrument is designed to help get a better understanding of how to apply and integrate new technologies and complex interventions in health care (Finch et al., 2015)**.**
This survey asks questions about the implementation of SDM via PHR. We understand that people involved with SDM via PHR have different roles, and that people may have more than one role.
From the statements below please choose an option that best describes ***your main role*** in relation to SDM via PHR:

**I am a *patient, aged 18-24yrs,* responsible for self-management of diabetes**	☐
**I am a *healthcare provider* responsible for the care of diabetic patients using electronic health record systems**	☐
**I am an *organizational provider* responsible for the design, development, implementation or management of electronic health record systems**	☐

For this survey, please answer all the statements from the perspective of this role. Depending on your role or responsibilities in SDM via PHR, some statements may be more relevant than others.
The survey is in 4 sections comprising detailed questions about the implementation of SDM via PHR. For each statement, there is the option to agree or disagree with what is being asked **(OPTION A).** However, if you feel that the statement is <u>not relevant to you</u>, there are also options to tell us why **(OPTION B).**

Please take the time to decide which answer **<u>best suits your experience for each statement and tick the appropriate circle.</u>**

Detailed questions about the intervention

For each statement please select an answer that best suits your experience using Option A. If the statement is not relevant to you please select an answer from Option B.

Section 1		Option A					Option B		
		Strongly Agree	Agree	Neither agree nor disagree	Disagree	Strongly disagree	Not relevant to my role	Not relevant at this stage	Not relevant to the intervention
1.	I can see how an integrated SDM via PHR system would differ from usual ways of working	○	○	○	○	○	○	○	○
2.	Patients and care providers at my clinic/ organization would have a shared understanding of the purpose of an integrated SDM via PHR system	○	○	○	○	○	○	○	○
3.	I understand how an integrated SDM via PHR system would affect the nature of my own work	○	○	○	○	○	○	○	○
4.	I can see the potential value of an integrated SDM via PHR system for my work	○	○	○	○	○	○	○	○

For each statement please select an answer that best suits your experience using Option A. If the statement is not relevant to you please select an answer from Option B.

Section 2	Option A					Option B		
	Strongly Agree	Agree	Neither agree nor disagree	Disagree	Strongly disagree	Not relevant to my role	Not relevant at this stage	Not relevant to the intervention
1. There are key people who would drive an integrated SDM via PHR system forward and get others involved	○	○	○	○	○	○	○	○
2. I believe that participating in an integrated SDM via PHR system is a legitimate part of my role	○	○	○	○	○	○	○	○
3. I would be open to working with patients/ care providers in new ways to use an integrated SDM via PHR system	○	○	○	○	○	○	○	○
4. I would continue to support an integrated SDM via PHR system	○	○	○	○	○	○	○	○

For each statement please select an answer that best suits your experience using Option A. If the statement is not relevant to you please select an answer from Option B.

Section 3	Option A					Option B		
	Strongly Agree	Agree	Neither agree nor disagree	Disagree	Strongly disagree	Not relevant to my role	Not relevant at this stage	Not relevant to the intervention
1. I could easily intercorporate an integrated SDM via PHR system into my existing work	○	○	○	○	○	○	○	○
2. An integrated SDM via PHR system would disrupt working relationships	○	○	○	○	○	○	○	○
3. I would have confidence in other people's ability to use an integrated SDM via PHR system	○	○	○	○	○	○	○	○
4. Work would be assigned to those with skills appropriate to an integrated SDM via PHR system	○	○	○	○	○	○	○	○
5. Sufficient training would be provided to enable patients and care providers to implement an integrated SDM via PHR system	○	○	○	○	○	○	○	○

**6. Sufficient resources would be available to support an integrated SDM via PHR**	○	○	○	○	○	○	○	○
**7. Clinic/ Organization management would adequately support an integrated SDM via PHR system**	○	○	○	○	○	○	○	○

For each statement please select an answer that best suits your experience using Option A. If the statement is not relevant to you please select an answer from Option B.

Section 4		Option A					Option B		
		Strongly Agree	Agree	Neither agree nor disagree	Disagree	Strongly disagree	Not relevant to my role	Not relevant at this stage	Not relevant to the intervention
1.	I would be aware of reports about the effects of an integrated SDM via PHR system	○	○	○	○	○	○	○	○
2.	Patients and care providers would agree that an integrated SDM via PHR system is worthwhile	○	○	○	○	○	○	○	○
3.	I value the effects that an integrated SDM via PHR system would have on my work	○	○	○	○	○	○	○	○
4.	Feedback about an integrated SDM via PHR system would be used to improve it in the future	○	○	○	○	○	○	○	○
5.	I would be able to modify how I work with an integrated SDM via PHR system	○	○	○	○	○	○	○	○
