# Supplementary material for: Ready for Prime Time? Using Normalization Process Theory to Evaluate Implementation Success of Personal Health Records Designed for Decision Making
Source: Front Digit Health. 2020 Nov 20;2:575951. doi: 10.3389/fdgth.2020.575951 (PMC8521962; doi:10.3389/fdgth.2020.575951)
Supplement: Supplementary Material 2 — The NoMAD: a measurement instrument of NPT. [file Data_Sheet_2.PDF]

# Supplementary Material 2: Practice-related Outcomes Survey

**This short survey is designed to better understand the potential outcomes from the integration of SDM via PHR in health care.**

Depending on your role or responsibilities in SDM via PHR, some statements may be more relevant than others.
For each statement, there is the option to agree or disagree with what is being asked **(OPTION A).** However, if you feel that the statement is not relevant to you, there are also options to tell us why **(OPTION B).**

Please take the time to decide which answer best suits your experience for each statement and tick the appropriate circle.

## Questions about outcomes of the intervention

**For each statement please select an answer that best suits your experience using Option A. If the statement is not relevant to you please select an answer from Option B.**

| | | Option A | | | | | Option B | | |
|---|---|---|---|---|---|---|---|---|---|
| | | Strongly Agree | Agree | Neither agree nor disagree | Disagree | Strongly disagree | Not relevant to my role | Not relevant at this stage | Not relevant to the intervention |
| 1. | An integrated SDM via PHR system would positively impact engagement in self-management decision making | ○ | ○ | ○ | ○ | ○ | ○ | ○ | ○ |
| 2. | An integrated SDM via PHR system would make it easier to participate in shared decision making | ○ | ○ | ○ | ○ | ○ | ○ | ○ | ○ |
| 3. | An integrated SDM via PHR system would reduce diabetes complications | ○ | ○ | ○ | ○ | ○ | ○ | ○ | ○ |
| 4. | An integrated SDM via PHR system would become a normal part of my work | ○ | ○ | ○ | ○ | ○ | ○ | ○ | ○ |
| 5. | An integrated SDM via PHR system would make it easier to support patients in managing their own care | ○ | ○ | ○ | ○ | ○ | ○ | ○ | ○ |
