# Supplementary material for: Ready for Prime Time? Using Normalization Process Theory to Evaluate Implementation Success of Personal Health Records Designed for Decision Making
Source: Front Digit Health. 2020 Nov 20;2:575951. doi: 10.3389/fdgth.2020.575951 (PMC8521962; doi:10.3389/fdgth.2020.575951)
Supplement: Supplementary Material 3 — Survey of practice-related outcomes. [file Data_Sheet_3.PDF]

University of Victoria

Diabetes Action Canada

Supplementary Material 3: Study Information
Using Personal Health Record Technology for Shared Decision Making as Routine Practice for Diabetic Youth

Definitions

Shared decision making (SDM) is a collaborative process that allows patients and their care providers to make healthcare decisions together by considering the best available medical evidence and patient values, goals and preferences in order to identify the best strategy at a particular point in time. SDM is not about convincing the patient to follow the care provider's recommendation; nor is it about leaving a patient to decide on her/ his own.

Personal health record (PHR) technology is an electronic health record application that allow patients to access, monitor, input, manage and share their health data and information. The application also gives patients access to personalized education materials, decision-support tools, and online communication options with care providers.

The user-validated *e*-PHR functional model

To enable SDM, the PHR functionality needs to enable the following actions:

- recognize and acknowledge a decision is required → **Acknowledge**
- get and interpret the best available options → **Consider**
- explore patient values and preferences and incorporate those into the making of a decision → **Decide**
- place that decision into an actionable care plan and track outcomes of the decision → **Act**

The integrated SDM via PHR system [*e*-PHR] environment is based on the interconnected PHR architectural type which gathers and populates patient data from multiple health information systems; as well as shared services (decision support and communications tools) for both patients and care providers. The *e*-PHR system is contextualized in a 3-minute video [click here to view].

A previous study resulted in a user-validation functional model [Figure 1] for *e*-PHR. This study seeks to identify and describe the likelihood of and promoting factors that may lead to a successful implementation of an *e*-PHR system.

**SDM Core Elements**	**Acknowledge** (recognize decision needed and respond)	**Consider** (get and interpret alternatives)	**Decide** (interact to explore preferences and incorporate them into the making of the treatment decision)		**Act** (record decision, track outcomes, and self-manage health)
**Essential *e*-PHR Functions by Patient Activity**	• Initiate and track SDM using info button<br>• Receive intelligent alerts	• Receive personalized decision support resources (e.g. decision aid, virtual assistant)<br>• Elicit preference in context of a treatment decision	• Review specific health data/ information<br>• Authorize provider access to patient data	• Participate in a virtual consultation with provider<br>• Export/ share diabetes dashboard summary<br>• Send/ receive message to/ from provider	• Co-author diabetes care plan (shared 'living' document)
**Optional *e*-PHR Functions by Patient Activity**			• Review provider clinical notes/ annotated data in provider EMR<br>• Review educational resources/ diabetes care plan	• Participate in an interactive bulletin board<br>• Send/ receive message to/ from virtual diabetes support group/ networks	• Use structured templates for the collection of diabetes 'observations of daily living'<br>• Manually enter personal narratives (e.g. mood, goals, values) and pictures
**Foundational *e*-PHR Functions by Patient Activity**	• Receive customizable reminders		• Receive health data from all digital health systems<br>• Review diabetes dashboard summary	• Make electronic request for appointment (face-to face or virtual)<br>• Make electronic request for prescription renewal & completion of standard forms	• Auto-populate health data from patient devices and applications<br>• Manually enter health data
**PHR Core Functional Categories**	**Receive Decision-Support**		**Access Health Information**	**Communicate with Others**	**Record Health Information**

Figure 1: *e*-PHR Functional model for the integration of SDM via PHR
