# Supplementary material for: Ready for Prime Time? Using Normalization Process Theory to Evaluate Implementation Success of Personal Health Records Designed for Decision Making
Source: Front Digit Health. 2020 Nov 20;2:575951. doi: 10.3389/fdgth.2020.575951 (PMC8521962; doi:10.3389/fdgth.2020.575951)
Supplement: Supplementary Material 4 — Semistructured interview guide. [file Data_Sheet_4.PDF]

Supplementary Material 4: Interview Guide

Interviewer Instructions:

1. Introduce self and affiliation
2. Confirm identity (speaking to right person)
3. Confirm this is still a good time to talk (need a total of about 60 minutes including the survey and interview), if not reschedule
4. Confirm that they are at a computer with internet and access to email
5. Remind them that you will record the conversation for transcription purposes
6. Confirm (re-affirm) they received consent form. Use verbal script Appendix 1B
7. Obtain verbal consent (or re-affirm).
8. Remind them about the purpose of the call
9. Thank them for participating, acknowledge their opinions and experiences as valuable to us
10. Confirm that they have received an email with the online surveys link and instructions for completion and ask them to complete it now (offer to mute their line and to stay on the call).
11. Thank them for completing the surveys and begin the interview

This call is to help us **understand the potential of an integrated SDM-PHR system to integrate into routine practice**, as well as to identify related facilitators and barriers.
The implementation of such a system has the potential to change:

- expertise and actions of systems
- the context of processes carried out to achieve goals
- patient and provider behaviour and outcomes

This is the second of two phases of the research about how PHR technology can be designed and implemented to facilitate the process of SDM in a manner which confirms its integration in clinical practice.

1. **How would you describe the integrated SDM-PHR system and is it distinct from your current practice?**
   a. Potential prompts:
      i. How is it distinct?
      ii. Does it have a clear purpose for patients and providers?

2. **Do you believe patients and providers will see the value and importance of the integrated SDM-PHR system?**
   a. Potential prompts:
      i. What benefits will the intervention bring and to whom?
      ii. Are these benefits likely to be valued by potential users?

3. **Do you believe it's right to engage in the use of the integrated SDM-PHR system?**
   a. Potential prompts:
      i. Are the potential users likely to think it's a good idea?

4. **Will users be prepared to invest time, energy and work into the use of the integrated SDM-PHR system?**
    a. Potential prompts:
        i. Do you think users can sustain involvement in the use of the system?

5. **Does an integrated SDM-PHR system fit with existing skill sets and work practices?**
    a. Potential prompts:
        i. Does it affect roles and responsibilities or training needs?
        ii. Will the system be supported and resourced?
        iii. Do you think users will have confidence in the system?

6. **Will the integrated SDM-PHR system make people's work easier?**
    a. Potential prompts:
        i. Will it promote or impede people's work?
        ii. Will it impact the division of labour and resources? power and responsibility?

7. **What would you say about the likely effects on patients or healthcare providers and their work environment?**
    a. Potential prompts:
        i. Is it likely to be perceived as advantageous for them?
        ii. Will it be clear what effects the intervention has had once it has been in use for a while?

8. **Will the integrated SDM-PHR system positively impact engagement in self-management decision making?**
    a. Potential prompts:
        i. Will it make it easier to participate in shared decision making?
        ii. Will it make it easier to support patients in managing their own care?

9. **Is the integrated SDM-PHR system likely to reduce diabetes complications?**

10. **What would you want this research to know about the opportunities and challenges with respect to the incorporation of such a system into routine practice to support self-management decision making?**

11. **We've talked a lot today about your opinions of an integrated SDM-PHR system to embed in clinical practice. Is there anything else you want to add that we haven't covered?**

Following the discussion, the interviewer will summarize the main points from the discussion and thank the participant.
